# 2337: Doxorubicin exposure in vitro stimulates ROS production and directly suppresses cardiac fibroblast proliferation

**DOI:** 10.1017/cts.2017.41

**Published:** 2018-05-10

**Authors:** Trevi A. Mancilla, Gregory J. Aune

**Affiliations:** University of Texas Health Science Center, San Antonio, TX, USA

## Abstract

OBJECTIVES/SPECIFIC AIMS: Our research strives to understand the pathophysiology of doxorubicin cardiotoxicity, focusing on the understudied nonmyocyte cardiac cells. Our understanding will enable researchers to develop protective or alternative therapies for cancer patients and treatments for cancer survivors. METHODS/STUDY POPULATION: Early studies have been carried out in isolated primary cardiac fibroblasts. Cells were treated with varying doses of doxorubicin. Cell viability, proliferation, and reactive oxygen species generation have all been studied. Future studies will focus on mitochondrial assessment in treated cells and confirmation of findings in animal models. Potential therapies discovered in these studies will also be conducted in animal models. RESULTS/ANTICIPATED RESULTS: Our results show a direct effect of doxorubicin on cardiac fibroblasts in vitro. Treated cells show a decreased rate of proliferation and increased production of reactive oxygen species. Similarly to cardiomyocytes, we hypothesize that reactive oxygen species damage the mitochondria of cardiac fibroblasts thereby altering their function and playing a role in doxorubicin cardiotoxicity. DISCUSSION/SIGNIFICANCE OF IMPACT: Current therapies have not been able to adequately protect patients from the cardiotoxicity of doxorubicin and other anthracyclines. A complete understanding of how doxorubicin damages cardiac tissue will only be possible by studying all cell types of the heart. With a better understanding, alternative therapies can be developed to prevent or treat doxorubicin cardiotoxicity without sacrificing the efficacy of doxorubicin in treating cancer.

